# Supplementary material for: Performance management and development system in South Africa, a necessary evil: Qualitative study
Source: PLoS One. 2025 Jul 1;20(7):e0317942. doi: 10.1371/journal.pone.0317942 (PMC12212554; doi:10.1371/journal.pone.0317942)
Supplement: S1 File — (PDF) [file pone.0317942.s001.pdf]

GAUTENG PROVINCE
HEALTH
REPUBLIC OF SOUTH AFRICA

**Performance Cycle-2022/23**	**Employees (Salary level 1-12)**

**Name and Surname:**		**Institution**	**GDOH**	**Component:**	**Ekurhuleni health district**
**Job Title:**		**Salary Level:**		**PERSAL Number:**	
**Health Facility** (main place of work)					
**Job Purpose:**					
**Name and Surname of Supervisor:**		**Supervisor PERSAL Number:**			
**Date of appointment:**		**Probation? Yes/No**			

KEY RESPONSIBILITY AREAS **(This section is specific to the official and could include projects)**

KEY RESPONSIBILITY AREAS (based on GDOH goals)	WEIGHTING (%)
OUTCOME 1: QUALITY OF HEALTH SERVICES IN PUBLIC HEALTH FACILITIES IMPROVED	30%
OUTCOME 2: MORBIDITY AND MORTALITY FROM COMMUNICABLE AND NON-COMMUNICABLE DISEASES REDUCED	30%
OUTCOME 3: PACKAGE OF SERVICES AVAILABLE TO THE POPULATION WITH PRIORITY GIVEN TO EQUITY AND MOST COST-EFFECTIVE SERVICES	30%
OUTCOME 4: LEADERSHIP AND GOVERNANCE IN THE HEALTH SECTOR ENHANCED TO IMPROVE QUALITY OF CARE	10%
	100%

OUTCOME 1: QUALITY OF HEALTH SERVICES IN PUBLIC HEALTH FACILITIES IMPROVED. 30%

STRATEGIC OBJECTIVE	OUTPUT	ACTIVITIES	OUTPUT INDICATOR	ANNUAL TARGET	ANNUAL TARGET	MILESTONE / QUARTERLY TARGETS 2022/23			
				2021/22	2022/23	Q1	Q2	Q3	Q4
1) To improve Maternal, child and women's health (MCWH)	Improved health of pregnant clients resulting in safe deliveries.	❖ Attend training of doctors and nurses on Basic antenatal care Plus (BANC Plus) guidelines.<br>❖ In-service training of nurses on Basic antenatal care Plus (BANC Plus) guidelines.<br>❖ Antenatal consultation by doctors at the CHCs and big clinics within 2-6 months of booking.<br>❖ Perform sonar for all booked pregnant patients.<br>❖ Detect and refer abnormalities in pregnancy.<br>❖ Organize / co-ordinate ESMOE drills for staff within the MOU at CHCs / PHC clinics.<br>❖ Attend mortality and morbidity reviews when nominated.	Percentage of antenatal consultation/sonars done for booked patients in your base clinic per quarter.	75%	80%	60%	65%	70%	80%

2)	To improve Paediatric care services in PHC facilities.	Improved clinical care of infants and the under 5 children.	❖ Train health workers on resuscitation of neonates.<br>❖ Training in management of common Paediatric conditions.<br>❖ Full implementation of IMCI at your base clinic.<br>❖ Provision of neonatal resuscitation protocol/guidelines in MOUs / PHC clinics	Percentage of Paediatric patients receiving appropriate care and according to IMCI guidelines in your base clinic per quarter.	80%	85%	70%	72%	80%	85%

OUTCOME 2: MORBIDITY AND MORTALITY FROM COMMUNICABLE AND NON-COMMUNICABLE DISEASES REDUCED. 30%									
**STRATEGIC OBJECTIVE**	**OUTPUT**	**ACTIVITIES**	**OUTPUT INDICATOR**	**Annual target**	**Annual target**	**Milestone / quarterly targets 2022/23**			
				**2021/22**	**2022/23**	**Q1**	**Q2**	**Q3**	**Q4**
1) To improve the management of Diabetic and hypertensive patients.	Improved control of patients' blood sugar and blood pressure.	❖ Calculate body mass index (BMI) of all patients consulted in the clinics.<br>❖ Review and educate patients on implications of abnormal weight gains.<br>❖ Implement lifestyle changes as required.<br>❖ Screening at risk population for diabetes and hypertension at all consultations.<br>❖ Ensure the use of hypertension and diabetes management guidelines.<br>❖ Review and ensure appropriate/rational use of hypertensive and diabetic medications.<br>❖ Ensure that annual monitoring parameters are done and addressed for hypertensive and diabetic patients	Percentage of Hypertensive and diabetic patients with their blood pressure and blood sugar levels controlled per quarter at your base clinic.	70%	75%	60%	65%	70%	75%

2) To improve the detection and management of mental health patients.	Improved identification, management, and timely referral of patients with mental health illness.	❖ Screen for mental health of all clients in all facilities as a component of consultation.<br>❖ Support sisters in the follow-up of Mental Health Care users	Percentage of patients screened for mental health conditions attending your base clinic per quarter.	50%	55%	30%	35%	45%	55%
3) To provide care, treatment and support for people living with HIV	Improved detection and management of People Living with HIV (PLHIV).	❖ Attend training for doctors in HIV /TB/STI management.<br>❖ Organize in-service for nurses on identified areas in HIV management.<br>❖ Provide and record Physician Initiated Counselling and Testing (PICT) by the doctor for all patients during consultations.<br>❖ Identify and manage patients with non-suppressed viral load.	Percentage of PLHIV consulted with viral load suppressed at base clinic per quarter.	55%	60%	40%	45%	50%	60%
4) To provide care, treatment and support for people suffering from TB	Improved detection and management of TB patients.	❖ Attend and support the quarterly HAST / TB review meetings when nominated.<br>❖ Screen for TB among patients consulted.	Percentage of patients screened for TB and put on treatment per quarter at your base clinic.	80%	85%	70%	75%	80%	85%

OUTCOME 3: PACKAGE OF SERVICES AVAILABLE TO THE POPULATION WITH PRIORITY GIVEN TO EQUITY AND MOST COST-EFFECTIVE SERVICES. 20%									
**STRATEGIC OBJECTIVE**	**OUTPUT**	**ACTIVITIES**	**OUTPUT INDICATOR**	**Annual target**	**Annual target**	**Milestone / quarterly targets 2022/23**			
**1)** To improve Clinical knowledge and skills of HCWs through in-service training.	Improved clinical knowledge and skills of health workers in the district.	❖ Attend CME/CPD meeting/workshops on common medical conditions at the district/ sub-district / facility for doctors /health care workers.<br>❖ Conduct/coordinate skills development /in-service training for HCWs at your base clinic at least once a month.<br>❖ Ensure patient consultation follow the SOAP approach and standard guidelines use<br>❖ Co-ordinate / attend morbidity and mortality meetings (MMM) / patient safety meetings (PSM) for health workers at the district/subdistrict/facility level at least once a quarter.	Percentage of patients managed with appropriate clinical knowledge and skills in your base clinic per quarter (SOAP approach and use of guidelines).	80%	85%	60%	70%	80%	85%

**2)**	To enhance accessibility of clinical services to poorest wards and disadvantaged communities in the district.	Improved quality of clinical health care services in communities and clinics	❖ Available to do outreach to any of the small clinics<br>❖ Use of approved treatment guidelines.<br>❖ Implement good consultations process.<br>❖ Maintenance of manual or electronic guidelines<br>❖ Organize meetings / in-service of WBOT at your base clinics.<br>❖ Provide clinical support to the WBOT teams.<br>❖ Ensure the clinic doctor and nurses refer all patients eligible for referral to the hospital appropriately.<br>❖ Maintain a referral book for all patients referred to hospital from clinics.	Percentage reduction in inappropriate referral of patients to the hospital from clinic base.	40%	50%	20%	30%	40%	50%

**3)** To maintain and improve 24 hours' clinical services in all community health centers (CHCs) in the district.	Improved and sustained accessibility of clinical services after-hours in all CHCs in the district.	❖ Available to participate in the overtime duties monthly.<br>❖ Manage your call duties adequately by arranging a swop when rostered and unable to attend your call duty.<br>❖ Sign call duty attendance register when on duty.	Percentage of appropriately signed call duty attendance register per quarter.	90%	95%	80%	85%	90%	95%
**4)** Improve services for older persons and disadvantaged individuals in the district.	Improved health and wellbeing of older persons and vulnerable groups in the district.	❖ Assign dedicated doctors to provide clinical services at old age homes and homes for the less privileged individuals/abused.<br>❖ Full medical examination of older persons with chronic medical conditions annually.	Percentage of older persons with chronic medical conditions with annual medical check-ups done per annum.	80%	85%	-	-	-	85%
**5)** To improve PHC medical and surgical emergency services in PHC facilities.	Improved survival of patients with life threatening medical conditions.	❖ Organise emergency lifesaving drills to maintain skills in the use of emergency equipment (e.g. defibrillator).<br>❖ Organize / coordinate / participate in training in emergency services such as BLS, ACLS, PALS and ATLS.<br>❖ Coordinate the maintenance of a well-organized emergency room trolley at the clinics daily.<br>❖ Perform minor surgical procedures such as suturing of minor lacerations, Incision & drainage of minor abscesses, splinting of simple fractures and removal foreign bodies at big clinics and CHCs.	Percentage of emergencies managed appropriately at your base clinic per quarter.	90%	95%	80%	85%	90%	95%

OUTCOME 4: LEADERSHIP AND GOVERNANCE IN THE HEALTH SECTOR ENHANCED TO IMPROVE QUALITY OF CARE. 20%									
**STRATEGIC OBJECTIVE**	**OUTPUT**	**ACTIVITIES**	**OUTPUT INDICATOR**	**ANNUAL TARGET**	**ANNUAL TARGET**	**MILESTONE / QUARTERLY TARGETS 2022/23**			
				**2021/22**	**2022/23**	**Q1**	**Q2**	**Q3**	**Q4**
1) To structure and improve medical training at the district.	Improved appropriate clinical knowledge and skills of interns allocated to the subdistrict.	❖ Supervise / mentor interns rotating through Family Medicine unit NSDR1.	Percentage of interns effectively and efficiently supervised / mentored per quarter.	90%	95%	-	-	-	95%

2) To improve work ethics among doctors at the clinics.	Improvement in the quality of clinical care services rendered at the PHC clinics.	❖ Daily signing of attendance registers on weekdays (medical doctors) appropriately.<br>❖ Adherence to clinically proven care of patients (SOAP approach and Flow chart).<br>❖ Rational use and appropriate combinations of medications.<br>❖ Appropriately fills in patients' prescription according to the expected standard.<br>❖ Trustworthiness / commitment / dedication to work of medical officer.<br>❖ Appropriately completes data collection documents (patient daily register)	Percentage effectiveness in the provision of quality clinical services at base clinic per quarter.	90%	95%	80%	85%	90%	95%

3) To improve administrative capacity.	Improved administrative and clinical governance role, knowledge, and skills of the medical officer.	❖ Attends meetings at base clinic monthly.<br>❖ Attends subdistrict doctors' meeting.<br>❖ Attends district doctors' meeting.<br>❖ Submits monthly report of base clinic on time (Friday of first week of each month).<br>❖ Submits sonar statistics of base clinic where applicable timeously (Friday of first week of each month).<br>❖ Ensure proper patient Booking system as recommended by ICSM program.<br>❖ Participate in monitoring, evaluation, and implementation of Ideal clinic recommendations at your base clinic.<br>❖ Available for prompt contracting / assessments - PMA, job description, quarterly and annual PMDS reviews.<br>❖ Punctuality/regularity at work.<br>❖ Carried out / participation in clinical	Percentage of objective clinical governance/management capacity of clinical services demonstrated per quarter.	80%	90%	75%	80%	85%	90%

		audit / QIP / Research							

ANNEXURE B

Section A

GENERIC ASSESSMENT FACTORS

KEY	Generic Assessment Factors (GAFs)	DEFINITION	APPLICABLE
	GAF1: Job knowledge	Knowledge of policies and practices	
	GAF2: Technical skills	Be able to use Technology	
	GAF3: Acceptance of responsibility	Accept responsibility for all work delegated to you	
	GAF4: Quality of work	Constantly produces exceptional work	
	GAF5: Reliability	Takes on additional work, no need to enquire about progress	
	GAF6: Initiative	High level of initiative, volunteers to do additional responsibilities no guidance needed	
	GAF7: Communication	Able to express facts and ideas clearly, able to write submissions etc.	
	GAF8: Interpersonal relationships	Cooperates well with supervisors and colleagues able to interact with others	
	GAF9: Flexibility	Ability to adapt to change in work routines, location etc.	
	GAF10: Teamwork	Able to work well with team members	
	GAF11: Planning and execution	Develop work plans evaluate them and achieve results	
	GAF12: Leadership	Motivate subordinates give and receive feedback resolve conflicts	
	GAF13: Delegation and empowerment	Need to provide clear responsibility to team. provide training and development opportunities.	
	GAF14: Management of financial GAF resources	Ability to control large volume of resources	
	GAF15: Management of human resources	Planned leave; Fair allocation of duties; sign payroll etc.	
	Total		

ANNEXURE C

Personal Development Plan

This section should be completed after employee and manager have agreed upon desired areas of improvement and development. Listed below are the suggested generic assessment factors from which only five may be selected. Add or delete GAFs suitable for specific requirements of the job. Then select two to three competencies for development and write a plan based on developmental objectives and the corresponding development activities (on-the-job training, formal training, and tertiary education); include measurements and time frames for completion.

Generic Assessment Factors		Competency Gaps (Tick)
1	Job knowledge	
2	Technical skills	
3	Acceptance of responsibility	
4	Quality of work	
5	Reliability	
6	Initiative	
7	Communication	
8	Interpersonal relationships	
9	Flexibility	
10	Teamwork	
11	Planning and execution	
12	Leadership	
13	Delegation and empowerment	
14	Management of financial resources	
15	Management of human resources	

Personal Development Plan					
**Key skills required**	**Intervention (Courses, Seminars, etc.<br><br>On-the job training**	**Training/ Development Programme Identified**	**Timelines**	**Desired Outcome for: -**	
				**Self**	**Department**

**Signature section:**	**Any disagreements will be dealt with in terms Regulation 72 (4) of the Public Service Regulation, 2016**

Names of Official: ______________________ **Signature:** ______________________

Date:

Agreed by Supervisor: ______________________ **Signature:** ______________________

Date:

Agreed by next level Manager: ______________________ **Signature:** ______________________

Date:
