# Supplementary material for: Performance management and development system in South Africa, a necessary evil: Qualitative study
Source: PLoS One. 2025 Jul 1;20(7):e0317942. doi: 10.1371/journal.pone.0317942 (PMC12212554; doi:10.1371/journal.pone.0317942)
Supplement: S2 File — (PDF) [file pone.0317942.s002.pdf]

MONTHLY PRACTICE SELF-ASSESSED PERFORMANCE AND MANAGEMENT TOOL

	**Performance Generic Dimensions Checklist**	**Comment/s**
**OUTCOME 1: QUALITY OF HEALTH SERVICES IN PUBLIC HEALTH FACILITIES IMPROVED**		
1.1<br>Reduce maternal morbidity and mortality.	a.<br>Total number of ANC patients seen by clinic in the quarter:<br>Total number of first visits in the quarter:<br>Total number of first visits 20 weeks and below:<br><br>Percentage of booked ANC patients for the doctor per quarter (Total number of patients booked for the doctor divided by total number seen by the doctor)<br><br>b.<br>Total number of sonars done < 20weeks per quarter<br>Total number of sonars 21 – 28 weeks per quarter<br>Total number > 28 weeks per quarter<br><br>Percentage of sonars done for booked pregnant women in the facility per quarter (Total number of patients booked for sonar divided by total number of sonars done.	
	ESMOE drills per quarter organized/participated per quarter.	
	Total number of successful maternal resuscitations done per quarter (source, MOU admissions/delivery register)	
1.2:<br>Reduce neonatal and child mortality and morbidity	Percentage of pediatric patients receiving appropriate care [treated according to IMCI guideline] <u>(Audit minimum of 20 files and attach audit page)</u>	
	Record of staff training on neonatal resuscitation	
	Number of successful neonatal resuscitations done per quarter (MOU admission/delivery register)	
1.3:<br>Patient experience of care	Patient satisfaction survey (source: operational manager's office)	
Outcome 1.4:<br>To ensure implementation of NHI	Your contribution to achieve ideal clinic status (source: operational managers office)	

programs		
**OUTCOME 2.1: IMPROVE MANAGEMENT OF NON-COMMUNICABLE DISEASES**		
2.1.1: Reduced prevalence of obesity among patients attending clinic	Percentage of patients seen with appropriate weight reduction measures implementation in your clinic per quarter (attach audit a minimum of 20 files)	
2.1.2: improved blood sugar control of diabetic patients	Percentage of controlled diabetic patients per quarter at your clinic (attach audit of a minimum of 20 files)	
2.1.3: Improved control of hypertensive patients	Percentage of controlled hypertensive patients per quarter at your (attach audit of a minimum of 20 files)	
2.1.4: Reduced complications in hypertensive patients	Percentage of controlled hypertensive patients with annual reviews and reflected on chronic flow chart (attach audit of a minimum of 20 files)	
2.1.5: Reduced complications in diabetic patients	Percentage of controlled diabetic patients with annual reviews and reflected on chronic flow chart (attach audit of a minimum of 20 files)	
2.1.6: Improved mental illness management	Percentage of patients screened for mental health conditions (attach audit of 10 to 15 files)	
**OUTCOME 2.2: IMPROVED MANAGEMENT OF COMMUNICABLE DISEASES**		
2.2.1: Improved management of PLHIV	Percentage of patients with appropriately filled HIV clinical record (attach audit of a minimum of 20 files of patients on HIV treatment)<br>Total number of patients on ART (TROA)<br>Total number of patients suppressed.<br>Total number with virological failure<br><br>Percentage suppression (total suppressed/total on ART X 100)	
2.2.1: Provide & support care of TB patients	Total head count<br>Total screened for TB<br>Total tested for TB.<br>Total tested positive<br>Total initiated on TB treatment.<br><br>Percentage of patients screened for TB in your clinic per quarter (Total screened for TB divided by total head count)	

**OUTCOME 3: PACKAGE OF SERVICES AVAILABLE TO THE POPULATION WITH PRIORITY GIVEN TO EQUITY AND MOST COST-EFFECTIVE SERVICES**		
3.1.1: Improve knowledge and clinical skills of doctors	Percentage attendance at subdistrict and district CMEs per quarter	
3.1.2: Improved patient care through appropriate consultation process, management and the maintenance of continuity of care	3.2 Percentage of patients managed with appropriate 3 stage assessment and plan per quarter (attach audit of a minimum of 20 files)	
3.1.3: Reduced waiting time of patients at the clinic	3.3 Percentage progressive reduction in waiting time per quarter at your clinic (obtain waiting time information from your DATA room)	
**OUTCOME 3.2: IMPROVED ACCESSIBILITY OF CLINICAL SERVICES TO POOREST WARDS AND DISADVANTAGED COMMUNITIES**		
3.2.2: Improved quality of clinical health care services in the small community clinics	Percentage of WBOT meetings / in-service training organized / attended per quarter (source: attendance registers)	
3.2.3: Improved referral of patients from clinics to hospitals	Percentage appropriate referral of PHC patients to hospital (source: referrals register)	
3.2.4: Improved and sustained accessibility of clinical services after hours in the CHCs	Percentage attendance to core duties and after hours (source: staff attendance register)	
**OUTCOME 3.3: IMPROVE EMERGENCY AND MINOR SURGICAL PROCEDURES AND SKILLS**		
3.3.1: Improved survival of patients with life threatening medical conditions	Percentage resuscitation of patients with life threatening medical conditions in your clinic per quarter (source: audit of resuscitation register)	
3.3.2: Reduce morbidity due to minor injuries at the clinic	Percentage of minor injuries / procedures successfully managed in allocated clinic per quarter (source: audit of procedure register)	

**OUTCOME 4.1: DEVELOP FUTURE CLINICAL LEADERS IN GAUTENG PROVINCE AND SOUTH AFRICA THROUGH FORMAL ACADEMIC TRAINING OF STUDENTS**		
4.1.1: Improved knowledge and skills of interns	Percentage of interns supported per quarter (source: record of interns rotation through the subdistrict)	
**OUTCOME 4.2: IMPROVED CONTRIBUTION TO INNOVATION AND DEVELOPMENT OF THE HEALTH SERVICES IN THE DISTRICT AND GAUTENG PROVINCE THROUGH RESEARCH**		
4.2: Improved innovative knowledge of clinical services in the subdistrict	Number of clinical audits / QIP organized / coordinated / participated in per quarter (source: clinical audits/QIP records)	
4.3.1: Improvement in the supervision of clinical care services at your clinic	Number of organized / coordinated / attended seminars/workshops to improve professional ethics / conducts per quarter (source: attendance registers/ provided feedbacks)	
4.3.2: Improved work productivity / performance of clinical staff within the clinic	Percentage participation in PMDS contracting and assessment (source: record of contracts signed with supervisor)	
**OUTCOME 4.4: IMPROVED ADMINISTRATION OF FAMILY MEDICINE UNIT WITHIN EKURHULENI HEALTH DISTRICT**		
4.4.1: Improved administrative and clinical governance role, knowledge, and skills of family physicians	Attend clinic meetings (attach attendance register)<br>Submit quarterly reviews on time.<br>Carry out assigned management duties appropriately.<br>Punctual and regular at work (appropriately signs attendance register)<br>Dedication / commitment at work<br><br>(self-assessment and record of supervisor’s clinic visits)	
