# Supplementary material for: Performance management and development system in South Africa, a necessary evil: Qualitative study
Source: PLoS One. 2025 Jul 1;20(7):e0317942. doi: 10.1371/journal.pone.0317942 (PMC12212554; doi:10.1371/journal.pone.0317942)
Supplement: S3 File — (PDF) [file pone.0317942.s003.pdf]

SEMI-STRUCTURED INTERVIEW GUIDE

TITLE: PERFORMANCE MANAGEMENT AND DEVELOPMENT SYSTEM IN SOUTH AFRICA: DOCTORS' EXPERIENCES. A QUALITATIVE STUDY.

1. You have all participated in the Performance Management Assessment. What do you know about performance assessment? Talk me through it.

2. Can you tell me what you feel or how you feel about it?

2. Monthly/quarterly self-assessment tools have been provided to monitor and manage your clinic. Have you found them useful in any way?

3. Has the performance assessment helped you in any way? Can you explain?

4. Has the performance assessment helped you know yourself or grow professionally? Can you explain?

5. Has the performance assessment helped you understand your roles and responsibilities in any way? Can you explain?

6. Has the performance assessment helped you improve the way you work? Can you explain?

7. Do you think performance assessment is useful for public service?

8. Do you think performance assessment is a good or fair way to assess public servants' performance for remuneration? Can you explain?

9. Any last comments?
